# Metal-organic frameworks in pharmaceutical research

**DOI:** 10.1016/j.pscia.2025.100096

**Published:** 2025-10-02

**Authors:** Zimeng Tao, Kun Hu, Baoxi Zhang, Shiying Yang, Dezhi Yang, Zhehui Zhao, Li Zhang, Yang Lu

**Affiliations:** a Beijing Key Laboratory of Innovative Drug Discovery and Polymorphic Research for Cerebrovascular Diseases, Institute of Materia Medica, Chinese Academy of Medical Sciences and Peking Union Medical College, Beijing, 100050, China; b State Key Laboratory of Bioactive Substance and Function of Natural Medicines, Beijing Key Laboratory of Active Substances Discovery and Drugability Evaluation, Institute of Materia Medica, Chinese Academy of Medical Sciences and Peking Union Medical College, Beijing, 100050, China

**Keywords:** Metal-organic framework, Drugs, Drug delivery system, Structural analysis

## Abstract

Metal-organic frameworks (MOFs) have emerged as a highly versatile class of porous materials with significant potential to advance pharmaceutical research. This review provides a comprehensive overview of the current landscape of MOFs, encompassing their synthesis strategies, characterization methodologies, and diverse biomedical applications. We detail various synthesis approaches (e.g., hydrothermal, electrochemical, microwave) and essential characterization techniques (e.g., X-ray diffraction (XRD), scanning electron microscopy (SEM), Brunauer-Emmett-Teller (BET) surface area analysis) that are critical for developing well-defined MOF structures. The review highlights the key advantages of MOFs in drug delivery, including their exceptional drug loading capacity, good biocompatibility, and capabilities for sustained, controlled, and targeted release. Their applications in improving drug solubility and stability, enabling pulmonary delivery, and functioning in biosensing, antimicrobial therapy, and nucleic acid delivery are also extensively discussed. Furthermore, we explore the utility of MOFs in drug structure analysis and the development of advanced functional systems, such as stimuli-responsive and self-propelled MOFs. Despite promising preclinical progress, challenges related to scalability, reproducibility, and long-term biosafety remain to be addressed for successful clinical translation. This work aims to bridge the gap between MOF materials science and pharmaceutical applications, offering valuable insights for the rational design of next-generation drug delivery systems and therapeutic platforms.

## Graphical abstract

**Figure:**
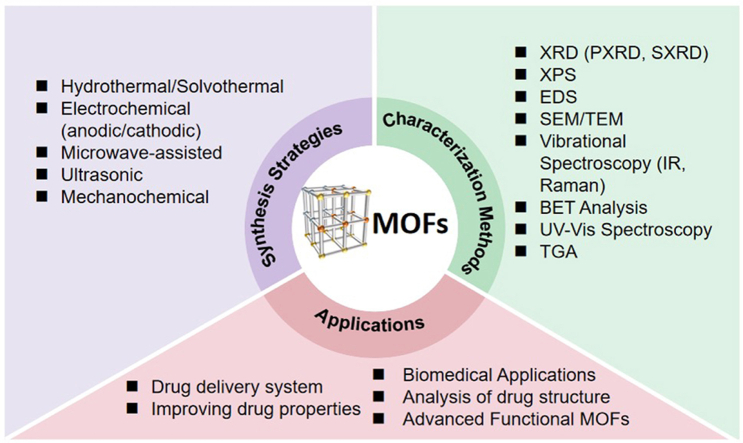

## 1 Introduction

MOFs are supramolecular compounds with one-to-three-dimensional periodic structures formed by the coordination between organic ligands and metal ions or metal cluster-based secondary building units (SBUs) through specific coordination sites [[1], [2], [3]].

The concept of MOFs was formally proposed by the research team of Professor Omar M. Yaghi from the University of California, Berkeley in 1995. They reported a coordination compound with a two-dimensional structure synthesized from the rigid organic ligand terephthalic acid and the transition metal Co, and named it the MOF [4]. In the mid-1990s, the Kitagawa research group in Japan synthesized the first-generation MOFs, but at this time, the pore structure of MOFs materials still needed the support of guest molecules, and the pore size and stability were limited [5,6]. In 1999, Yaghi's group designed MOF-5, a metal-organic framework material composed of the rigid organic ligand terephthalic acid and a transition metal Zn. It has become a milestone in the research of metal-organic framework materials. It has high porosity and good gas adsorption performance, and the skeleton is still intact after the guest molecules are removed from the pore, showing the great potential of MOFs in gas adsorption [7]. In 2004, the research group of Gerard Ferey at the University of Versailles in France reported a molecular sieve type MOF with large pores, MIL-100 [8]. In 2006, Yaghi's group turned its attention to traditional molecular sieve materials with superior stability properties, and synthesized 12 molecular sieve imidazole backbone materials, ZIF-1 to ZIF-12, with 7 typical silica-aluminum molecular sieve topologies. These materials exhibit superior thermal and chemical stability [9]. In the subsequent series of studies, functional MOFs such as the UiO series [[10], [11], [12]] and the NU series [[13], [14], [15]] were reported, while the MIL series [16,17] and the ZIF series [18,19] continued to be developed. Compared with traditional porous solid materials (e.g., zeolites and carbon-based porous materials), MOFs exhibit superior performance across various applications. This significant scientific potential has attracted growing interest from researchers worldwide.

This comprehensive review aims to systematically examine the current state and future prospects of MOFs in pharmaceutical research, with particular emphasis on their synthesis strategies, characterization methods, and diverse biomedical applications. The review follows a logical progression from fundamental synthesis approaches to advanced pharmaceutical applications, highlighting the unique advantages of MOFs including their exceptional tunability, high drug loading capacity, and multifunctional capabilities. The innovation of this work lies in providing a holistic perspective that bridges the gap between MOF materials science and pharmaceutical applications, offering insights into how structural design principles can be leveraged to address specific challenges in drug delivery, bioavailability enhancement, and therapeutic efficacy.

## 2 Synthesis strategies of MOFs

### 2.1 Hydrothermal/Solvothermal synthesis method

The hydrothermal or solvothermal method is the most commonly used approach to synthesize MOFs, which have the advantages of precise control over the product's particle size, shape, and crystallinity [20]. However, the solvothermal method has many disadvantages, such as long reaction time, up to several hours or even days. The reaction conditions are more stringent, usually requiring a high-temperature and high-pressure environment. The consumption of organic solvents is large and easy to cause environmental pollution [21]. The reaction of the original mixture is carried out in a closed system with water or liquid organic solvents as the solvent at a certain temperature and under the autogenic pressure of the solution. In this way, MOF microcrystalline products can be easily obtained under heating conditions, and even single crystal products suitable for single crystal resolution can be obtained. MOF-5 synthesized by Yaghi research group using terephthalic acid was synthesized by solvothermal synthesis [7]. Sun et al. used the solvothermal method to prepare MOF-derived pure phase ZnO and ZnO/Co_3_O_4_ composite microstructures with different ratios [22]. While these methods are foundational, their inefficiencies highlight the need for greener and more scalable alternatives.

### 2.2 Electrochemical synthesis method

Electrochemical synthesis of MOFs encompasses two primary mechanisms: anodic dissolution and cathodic deposition. This approach offers distinct advantages over conventional solvothermal methods, including potentially faster reaction times, better control over film formation on electrodes, and enhanced scalability. In anodic dissolution, the applied potential oxidizes the metal electrode, releasing ions that subsequently coordinate with organic linkers in the solution to form MOFs. Conversely, cathodic deposition relies on the electrochemical reduction of species (like protons or nitrate ions) at the cathode surface, generating a localized pH increase that triggers deprotonation of linkers and precipitation of MOFs from solution containing pre-dissolved metal salts. The significance of electrochemical routes was highlighted when BASF pioneered the concept in a 2005 patent, utilizing anodic dissolution. Their setup employed copper plates as both anode and cathode in a methanol solution containing trimesic acid (H_3_BTC), applying 12–19 ​V to achieve a current of 1.3 A for 150 ​min, resulting in the successful synthesis of Cu-MOF (HKUST-1) as a blue precipitate [23]. This demonstration spurred wider exploration of the technique's versatility. Following this, Martinez et al. significantly expanded the scope by electrochemically synthesizing not only HKUST-1 but also diverse frameworks including ZIF-8, Al-MIL-100, Al-MIL-53, and Al-MIL-53-NH_2_ using anodic methods [24], underscoring the methodology's adaptability to various metal ions (Cu, Zn, Al) and linker systems, and its potential as a general platform for MOF fabrication.

### 2.3 Microwave synthesis method

Microwave synthesis operates by coupling an oscillating electric field with the permanent dipole moments of molecules in the reaction medium, inducing rapid molecular rotation and efficient heating of the liquid phase [23]. This method is particularly effective for the preparation of MOFs, yielding materials with high phase purity and suitability for obtaining small-sized crystals. It is widely acknowledged that microwave irradiation enhances reaction kinetics and promotes homogeneous nucleation, thereby contributing to improved crystallinity and reduced particle size dispersity. Key advantages of this technique include accelerated crystallization rates, enhanced phase selectivity, narrow particle size distributions, and facile morphology control [[25], [26], [27], [28], [29]]. The first successful application of microwave synthesis to MOFs was demonstrated in the preparation of MIL-100 (Cr), which was synthesized at 220 ​°C within only 4 ​h (yield: 44%)—a synthesis time significantly shorter than the 4 days required by conventional hydrothermal methods [30]. Since then, various MOF structures such as Fe-MIL-53 [31], Fe-MIL-101-NH_2_ [32], IRMOF-3 (H_2_BDC-NH_2_) [33] and ZIF-8 (2-ethylimidazole,HMeIm) [34] have been efficiently synthesized via microwave-assisted routes.

### 2.4 Ultrasonic method

The raw material is dissolved in a solvent and subjected to continuous ultrasonic treatment. This method utilizes ultrasonic energy to promote the persistent generation of bubbles in the solvent, achieving uniform nucleation of MOFs through the dynamic processes of bubble formation, growth, and violent collapse. It is generally recognized that the extreme local temperatures and pressures generated during cavitation significantly enhance mass transfer and reaction kinetics, thereby promoting rapid and homogeneous crystallization. As a result, this process substantially shortens the crystallization time and facilitates the formation of crystals with smaller sizes [23]. Compared to conventional solvothermal synthesis, ultrasonication not only reduces the crystallization duration but also leads to a notable decrease in crystal particle size [35,36]. For instance, Son et al. employed 1-methyl-2-pyrrolidone (NMP) as a solvent and successfully obtained MOF-5 crystals with sizes ranging from 5 to 25 ​μm within 30 ​min via ultrasonic synthesis [37]. Cho et al. utilized NaOH and triethylamine (TEA) as additives to prepare ZIF-8 efficiently using an ultrasonic method [38].

### 2.5 Mechanochemical synthesis

Mechanical forces are capable of inducing a variety of physical phenomena and chemical reactions [39]. In mechanochemical synthesis, these forces facilitate the breaking of intramolecular bonds, which is followed by chemical transformation into new products [40]. A significant advantage of this approach is its ability to drive reactions through mechanical energy input rather than thermal activation, offering a distinct pathway for material synthesis. Notably, mechanochemical reactions can often be performed at room temperature and in the absence of solvent [41], which substantially shortens reaction time, eliminates the need for high temperatures, and facilitates large-scale, high-throughput synthesis of MOFs [[42], [43], [44]]. Pichon et al. successfully synthesized HKUST-1 via a solvent-free mechanochemical route [45]. Beldon et al. utilized ZnO with imidazole (HIm), 2-methylimidazole (HMeIm), and 2-ethylimidazole (HEtIm) as starting materials to synthesize various ZIFs within 30–60 ​min using mechanochemical methods [46]. The overview of common synthesis methods for MOFs is shown in Table 1.

**Table 1:** Overview of common synthesis methods for MOFs.

Method	Principle	Key Advantages	Limitations	Representative MOFs Synthesized
Hydrothermal/Solvothermal	Reaction in closed system with water/organic solvent at elevated temperature and autogenous pressure.	Precise control over particle size, shape, and crystallinity; suitable for single crystal growth.	Long reaction time (hours to days); requirement for high temperature/pressure; high solvent consumption.	MOF-5, ZIF series, MIL series
Electrochemical	Anodic dissolution or cathodic deposition under applied potential.	Faster reaction; controlled film formation; scalable; reduced solvent use.	Requires electrodes and conductive solutions; limited to certain metals.	HKUST-1, ZIF-8, Al-MIL-100, Al-MIL-53
Microwave	Dielectric heating via coupling of microwave energy with molecular dipoles.	Rapid crystallization; high phase purity; narrow size distribution; energy-efficient.	Limited to small batch sizes; requires specialized equipment.	MIL-100 (Cr), IRMOF-3, ZIF-8, Fe-MIL-101-NH_2_
Ultrasonic	Cavitation-induced nucleation via bubble formation and collapse.	Short reaction time; uniform nucleation; small crystal size.	Energy-intensive; may require additives for optimal results.	MOF-5, ZIF-8
Mechanochemical	Mechanical force-induced bond breaking and chemical transformation.	Solvent-free; room temperature; short reaction time; scalable and green.	Limited to certain ligands; may require milling media.	HKUST-1, ZIF-1, ZIF-8, ZIF-11

## 3 Methods for characterizing MOFs

The study of MOFs materials usually involves the analysis of multi-technical representations.

XRD is essential for the structural characterization of MOFs, and its two primary techniques are powder XRD (PXRD) and single-crystal XRD (SXRD). PXRD serves as a powerful tool for routine phase analysis and quality assessment, enabling researchers to determine crystal structure, assess sample purity, evaluate framework stability, and monitor reaction progress by comparing the positions and intensities of diffraction peaks with reference patterns [[47], [48], [49], [50]]. In contrast, SXRD provides atomic-resolution structural details and is employed to resolve complete crystal structures and obtain precise crystallographic data [[51], [52], [53]]. The utility of XRD extends beyond structural confirmation to probing functional behavior under realistic conditions. A compelling example is provided by Zhang et al., who used XRD to investigate the stability of ZIF-90 in solutions of varying pH (5.5–7.4). Their analysis revealed that ZIF-90 maintains high structural stability under neutral and basic conditions but undergoes significant degradation in acidic environments (pH 5–6), which simulate the extracellular conditions around tumor cells. This pH-responsive instability effectively demonstrates the potential of ZIF-90 as a smart carrier for targeted anticancer drug delivery [54].

X-ray photoelectron spectroscopy (XPS) is a highly surface-sensitive technique widely employed to analyze the chemical composition and electronic states of materials. It is particularly valuable for characterizing the near-surface region of MOFs, providing quantitative information about elemental composition, chemical bonding, and local chemical environments. This capability makes XPS indispensable for investigating surface functionalization, adsorption mechanisms, and chemical stability in MOF-based materials [[55], [56], [57]].

Energy dispersive spectroscopy (EDS) is a powerful analytical technique frequently coupled with electron microscopy for elemental characterization of materials. It is particularly useful for providing rapid semi-quantitative elemental composition mapping within MOF structures, enabling the analysis of elemental distribution, chemical homogeneity, and potential dopant incorporation [58]. Beyond basic composition, EDS can also offer insights into chemical environments through subtle shifts in peak positions, though its primary strength remains elemental identification and spatial distribution. For instance, Tse et al. employed a vector-based algorithm to analyze energy-dispersive X-ray spectra from representative particles, successfully assessing the relative abundance of oxygen (O), fluorine (F), and potassium (K) within their sample [55]. This approach highlights how advanced data processing of EDS results can yield detailed compositional information critical for understanding the local chemical structure of MOFs.

**Fig. 1:**
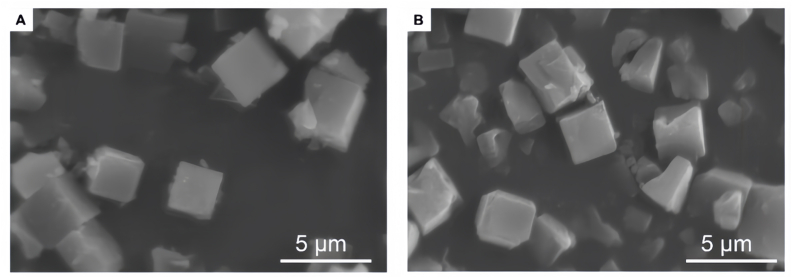
(A) SEM Image of γ-CD-MOF; (B) SEM Image of D-Lim@γ-CD-MOF. *Reprinted from Y. Zhou et al., International Journal of Pharmaceutics, 2021, 606, 120825, with permission from Elsevier*.

SEM and transmission electron microscopy (TEM) are indispensable techniques for characterizing the morphology and microstructure of MOFs. These methods provide critical insights into the relationship between synthetic conditions and the resulting macroscopic structure of MOFs, which directly influences their properties and performance. SEM is primarily used to examine surface topography, particle size, and overall shape, whereas TEM offers higher resolution, enabling the observation of crystal lattices, internal defects, and detailed structural features at near-atomic scales [47,51,56,59]. The capability of electron microscopy to correlate synthesis methods with morphology is well illustrated in the literature. For example, Tse et al. used SEM to reveal that cyclodextrin-based MOF (CD-MOF) particles synthesized via spray drying exhibited a depressed spherical morphology, whereas those prepared by antisolvent crystallization adopted a bulkier, irregular shape [55]. Zhou et al. employed SEM to confirm the uniform cubic morphology and narrow size distribution (1–5 ​μm) of γ-CD-MOF particles, and importantly, demonstrated that this well-defined shape was preserved even after the loading of D-limonene (D-Lim), indicating good structural stability (Fig. 1) [60].

Vibrational spectroscopy, encompassing both infrared (IR) and Raman spectroscopy, serves as a fundamental tool for probing the molecular structure, symmetry, chemical properties, and bond strengths of materials. A key advantage of these methods is their sensitivity to local chemical environments and bonding, offering a complementary perspective to diffraction-based structural analysis. When used in combination with other characterization techniques, IR and Raman spectroscopy can provide a comprehensive understanding of the structure, properties, and reactivity of MOFs [61]. Specifically, IR spectroscopy is highly effective for identifying functional groups and characterizing chemical bonds present in the organic ligands of MOFs, providing crucial evidence for successful synthesis and postsynthetic modification [[47], [48], [49],^53^,^60^].

BET analysis is a fundamental technique for characterizing the porosity of MOFs, enabling the determination of specific surface area, pore size distribution, and gas adsorption properties [53,56,59]. This method is indispensable for establishing structure-property relationships in porous materials, as it quantitatively links porosity to potential applications in gas storage, separation, and drug delivery. Commonly used probe gases include nitrogen, argon, carbon dioxide, and hydrogen. The power of BET analysis in elucidating pore accessibility and host-guest interactions is demonstrated in several studies. Chen et al. investigated the effect of loading isosteviol (STV) on the porosity of a CD-MOF by measuring its nitrogen adsorption capacity. They reported a BET surface area of 278.7 ​m^2^/g for the pristine CD-MOF, which drastically decreased to 6.6 ​m^2^/g after drug incorporation. This significant reduction indicates that the drug molecules occupied the majority of the pores within the framework, thereby hindering nitrogen adsorption and confirming successful pore loading [49]. Similarly, Nabipour et al. utilized BET analysis to explore the feasibility of drug storage within the pores of Zn_2_(bdc)_2_(dabco), further underscoring the utility of this technique in evaluating MOFs for drug delivery applications [47].

Ultraviolet–visible (UV–Vis) spectroscopy is a key technique for investigating the light absorption properties of MOFs and is extensively applied in the study of guest encapsulation and release processes [62]. Its primary strength lies in the quantitative monitoring of molecular concentrations, making it indispensable for evaluating drug loading efficiency and release kinetics in MOF-based delivery systems. This utility is well demonstrated in several drug delivery studies. Guo et al. employed UV–Vis spectroscopy to determine the concentration of benzotriazole (BTA) molecules released over specific time intervals, thereby verifying the sustained release behavior from BTA@MOF-5 [59]. In a separate study, Zhang et al. utilized UV–Vis to quantify the drug loading of verapamil (VER) and doxorubicin (DOX) in ZIF-8 nanoparticles. The measured loadings were approximately 8.9% for DOX and 32% for VER, values which indicate a superior loading capacity compared to many traditional drug delivery platforms [52]. Similarly, Tabatabaeian et al. applied UV–Vis spectroscopy to analyze the release profile of rosmarinic acid (RA) from a novel MOF-based carrier, further highlighting the technique's role in characterizing release dynamics [63].

Thermogravimetric analysis (TGA) was used to analyze the thermal stability of the scaffolds [48,59,64]. TGA can be used to study the thermal stability and decomposition process of MOF, and determine the thermal decomposition temperature and weight loss of MOF [47,52,53].

## 4 Application of MOFs in the field of pharmaceutical research

MOFs possess a unique set of properties, including exceptionally large surface areas, tunable pore sizes, and the capacity for functionalized modification, which make them highly versatile materials [[65], [66], [67]]. These structural and chemical features provide an exceptional platform for designing tailored materials with specific functionalities. Consequently, MOFs have found widespread applications across biological and medical fields [[68], [69], [70], [71], [72], [73], [74], [75], [76]]—such as drug delivery and biosensing—as well as in chemical and energy materials [67,[77], [78], [79], [80], [81], [82], [83], [84], [85], [86]]—including catalysis, gas storage, and separation. In particular, recent years have witnessed MOFs demonstrating significant potential and achieving notable progress in pharmaceutical research, where their high drug loading capacity and stimuli-responsive release profiles offer new strategies for advanced therapeutics.

### 4.1 Drug delivery system

#### 4.1.1 High drug loading

Owing to their high specific surface area, tunable pore size, exceptional porosity, and ease of functionalization, MOFs exhibit a drug loading capacity that significantly surpasses that of many conventional drug carriers, positioning them as ideal platforms for drug delivery applications [68,87,88]. The defining structural features of MOFs—namely their ultrahigh surface areas and modular porosity—provide abundant adsorption sites and vast internal space for the incorporation of therapeutic molecules, which directly enhances both drug loading and delivery efficiency.

This superior performance was early demonstrated by Horcajada et al., who reported the significant adsorption of ibuprofen on MIL-100 and MIL-101, thereby establishing the potential of MOFs as effective drug carriers [89]. The correlation between surface area and loading capacity is generally positive, a principle exemplified by the work of Farha et al., who synthesized NU-109 and NU-110, MOFs possessing record-breaking experimental BET surface areas and approaching a theoretical upper limit of 14,600 ​m^2^/g [90].

**Fig. 2:**
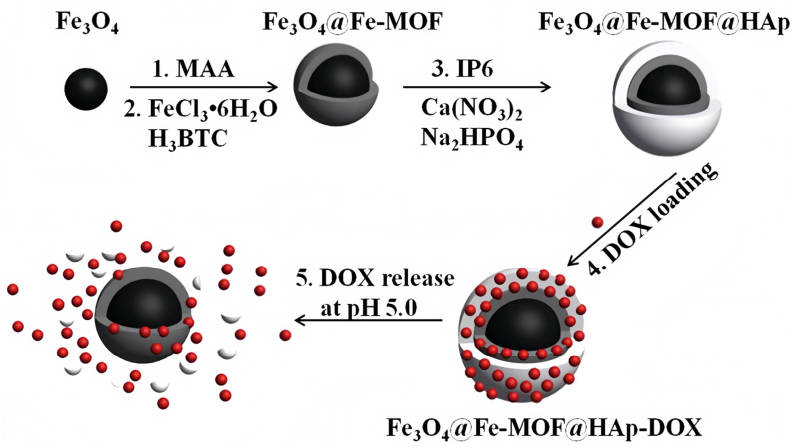
Schematic illustration of the preparation process of Fe_3_O_4_@Fe-MOF@HAp nanocomposites and their drug loading and release behavior. *Reproduced from Y. Yang* et al.*, Journal of Materials Chemistry B, 2017, 5, 8606, with permission from the Royal Society of Chemistry.*

Subsequent studies have further validated the exceptional loading performance of MOF-based systems. Yang et al. developed Fe_3_O_4_@Fe-MOF@HAp composite microspheres (Fig. 2), which achieved a high DOX loading of 75.38 mg/g—a capacity markedly superior to that of Fe_3_O_4_@mesoporous SiO_2_ (mSiO_2_) (12.3 ​mg/g) and selenium nanoparticles (17 ​mg/g) [91]. Similarly, Zhang et al. utilized nanoscale ZIF-90 for the co-delivery of the anticancer drugs 5-fluorouracil (5-FU) and DOX, achieving notable loadings of 36.35% and 11–13.5 ​wt%, respectively [54].

#### 4.1.2 Good biocompatibility

**Fig. 3:**
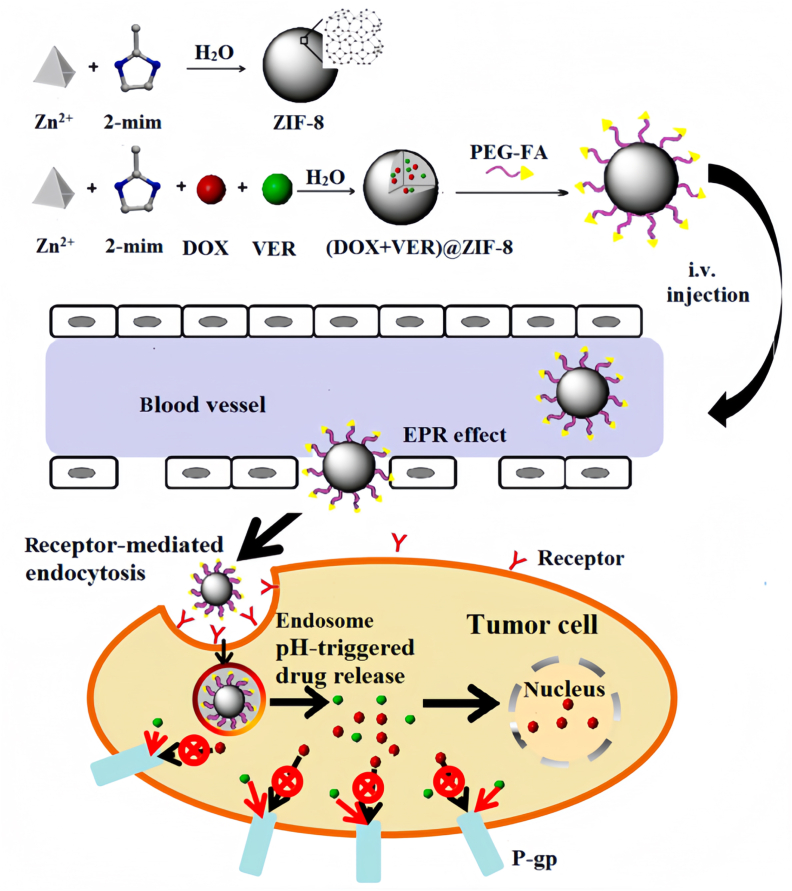
Schematic illustration of the preparation of PEG-FA-modified (DOX ​+ ​VER)@ZIF-8 nanoparticles and their mechanism of targeted delivery, cellular uptake, and pH-responsive drug release for overcoming multidrug resistance. *Reprinted with permission from H. Zhang* et al.*, ACS Applied Materials & Interfaces, 2017, 9(23), 19687–19697. Copyright 2017 American Chemical Society.*

When employing MOFs in biomedical applications, biocompatibility is a critical factor that must be carefully considered. Consequently, the rational selection of low-toxicity organic ligands and biocompatible metal ions is a fundamental strategy for constructing safe and effective MOF-based nanocarriers. This design principle is essential for minimizing adverse reactions and ensuring clinical translatability [60]. The success of this approach is demonstrated in several studies. Zhang et al. developed poly (ethylene glycol)-folate (PEG-FA) functionalized ZIF-8 nanoparticles for the tumor-targeted co-delivery of DOX and VER (Fig. 3). Their system demonstrated not only enhanced blood circulation but also a high degree of biosafety, underscoring the importance of surface modification in improving biocompatibility [92].

Similarly, Yang et al. conducted *in vitro* cytotoxicity assays which confirmed the good biocompatibility of MOF-5, suggesting its strong potential as a carrier for anticancer drugs [48].

#### 4.1.3 Sustained and controlled drug release

The pore architecture of MOFs can be precisely tuned by selecting specific metal centers and organic ligands, enabling the accommodation of drug molecules with varying sizes and physicochemical properties. This synthetic versatility allows for the rational design of MOFs to achieve high drug loading and programmable release profiles, which is a significant advantage over conventional delivery systems. By encapsulating therapeutics within the porous matrix, MOFs can facilitate sustained drug release, thereby prolonging the duration of pharmacological action and reducing dosing frequency.

The release kinetics can be further controlled through deliberate structural or surface modifications. For instance, Yang et al. investigated the *in vitro* release of capsaicin and 5-FU from MOF-5 using a dialysis bag method. They reported cumulative releases of 76.8% and 73.8%, respectively, demonstrating a clear sustained-release profile [48]. In a more advanced approach, Jiang et al. developed a PEG-coated anionic MOF system, which significantly enhanced stability and prevented premature drug release under gastric conditions, enabling efficient intestinal-specific delivery via strong host–guest interactions [93].

#### 4.1.4 Targeted drug delivery

MOFs can be engineered for targeted drug delivery through strategic surface modification and functionalization, thereby enhancing drug accumulation at the disease site and improving therapeutic efficacy. A key design principle involves incorporating stimuli-responsive elements or targeting moieties that exploit the pathological microenvironment—such as low pH, enzymes, or magnetic fields—to achieve spatiotemporally controlled drug release.

This approach is exemplified in several studies. Yang et al. developed Fe_3_O_4_@Fe-MOF@HAp composite microspheres, where the magnetic Fe_3_O_4_ core enables effective magnetic targeting, and the pH-sensitive hydroxyapatite (HAp) shell facilitates responsive drug release in the acidic tumor microenvironment. This design allows for the controllable release of DOX, significantly enhancing the anti-tumor effect [91]. Similarly, Zhang et al. demonstrated that nanoscale ZIF-90 enables cancer-targeted co-delivery of anticancer drugs. The framework remains stable under physiological conditions (extending 5-FU release to 25 ​h) but rapidly degrades in the acidic tumor environment (pH 5.5), releasing over 95% of the drug in less than 16 ​h, thereby ensuring selective and efficient drug release at the tumor site [54]. Further functionalization can enhance both targeting and therapeutic mechanisms. Chen et al. synthesized folic acid (FA)-functionalized and epigallocatechin-3-gallate palmitate (PEGCG)-modified ZIF-8 nanoparticles (PEG-FA/PEGCG@ZIF-8 NPs). This system not only serves as a pH-responsive carrier for targeted chemotherapy but also significantly induces autophagic flux and autophagosome formation in treated cells, showcasing a combined strategy for targeted and synergistic therapy [94].

#### 4.1.5 Pulmonary drug administration

MOFs exhibit a suite of highly suitable properties for serving as carriers in dry powder inhaler (DPI) formulations for pulmonary delivery, including low density, high porosity, and excellent biocompatibility. These characteristics collectively enhance drug deposition in the deep lung and promote subsequent absorption, making MOFs a promising platform for respiratory nanomedicine. Their tunable aerodynamic properties and ability to stabilize therapeutic agents further support their application in inhalable drug delivery.

**Fig. 4:**
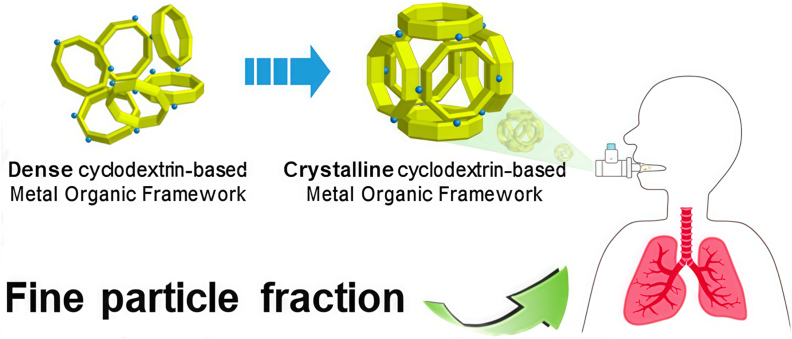
Schematic illustration of the transformation of dense CD-MOF into a crystalline form and its subsequent pulmonary delivery via inhalation. *Adapted with permission from J.Y. Tse* et al.*, Crystal Growth & Design, 2021, 21(2), 1143–1154. Copyright 2021 American Chemical Society.*

This potential is well illustrated in several studies. Tse et al. prepared CD-MOF particles via spray drying, which exhibited a uniform spherical morphology, low density, and a geometric median diameter (D_50_) below 5 ​μm—a critical size range for effective pulmonary deposition (Fig. 4). These properties confirm the strong potential of CD-MOFs as excipients in DPI formulations [55]. In a separate study, Zhou et al. loaded curcumin into CD-MOFs, significantly enhancing its solubility and achieving favorable aerodynamic performance. This system demonstrates the potential of CD-MOFs as carriers for improving the pulmonary delivery of poorly water-soluble drugs [95]. Furthermore, the same group successfully incorporated D-Lim into biodegradable γ-CD-MOF, which notably improved the stability of the volatile compound. This work underscores the importance of MOF-based systems in developing stable and effective DPIs [60].

### 4.2 Improving drug properties

MOFs offer a robust strategy to enhance the solubility, stability, and bioavailability of poorly performant drugs. This is particularly impactful for natural compounds, which often exhibit high therapeutic potential but are limited by poor aqueous solubility and low bioavailability; MOFs can effectively overcome these barriers through encapsulation and protection within their porous structures. For instance, the low solubility and bioavailability of STV, a natural compound, significantly restrict its clinical application. Chen et al. demonstrated that encapsulating STV in a CD-MOF effectively addressed these challenges. The bioavailability of STV was markedly higher in the STV@CD-MOF (1:1) complex than in a simple inclusion complex with the conventional cyclodextrin (CD), underscoring the advantage of the MOF-based carrier [49]. Similarly, Chen et al. showed that encapsulating PEGCG in ZIF-8 nanoparticles improved its stability by a factor of six compared to free PEGCG, thereby maintaining its integrity under physiological conditions [94]. Furthermore, CD-MOF has been employed to load 18β-glycyrrhetinic acid (GA), another natural product with poor solubility. This formulation significantly enhanced both the solubility and bioavailability of GA, leading to an improved therapeutic effect in a rat model of idiopathic pulmonary fibrosis (IPF) [50].

### 4.3 Biomedical applications: diagnostics and therapeutics

#### 4.3.1 Diagnostics

MOFs exhibit exceptional promise as “smart biotechnological tools” particularly for biosensing and antimicrobial applications, leveraging their high porosity, tunable pores, and structural versatility. This makes them highly attractive for addressing critical limitations in current diagnostic and therapeutic approaches. In virus detection, traditional methods like reverse transcription-polymerase chain reaction (RT-PCR), polymerase chain reaction (PCR), and serological assays, while established, are often time-consuming and require complex instrumentation and skilled operation [[96], [97], [98], [99]]. MOFs offer a compelling alternative pathway through fluorescent biosensing platforms [100]. Nanoscale MOFs can function as efficient platforms for nucleic acid detection via fluorescence, exemplified by Zhu et al.'s pioneering strategy using Cu(II)-MOF. This system adsorbed fluorescent probe DNA and released it upon target binding, inducing a measurable fluorescence change [101]. The versatility of this approach is further demonstrated by platforms like Tabatabaeian et al.'s green-fluorescent UiO-66-NH_2_@N-CNDs, which serve dual roles in drug delivery and cellular imaging [102], and the use of MIL-88 and MIL-101 frameworks for nucleic acid detection [[103], [104], [105]].

#### 4.3.2 Antimicrobial activity

MOFs possess significant intrinsic or engineered antimicrobial activity. Their antibacterial mechanisms primarily involve structural degradation leading to sustained release of bioactive metal ions and/or surface interactions [106]. This functionality can be harnessed in multiple ways: MOFs can act as intrinsic antimicrobial agents, serve as carriers for conventional antimicrobial drugs, or be combined with other nanomaterials [107]. A key advantage is their ability to store and controllably release antimicrobial payloads from their pores [100]. Sava et al. successfully encapsulated ceftazidime in ZIF-8, preserving drug activity with a 10.9% loading rate [108], while Nabipour et al. loaded gentamicin into Zn_2_(bdc)_2_(dabco), achieving pH-responsive controlled release (enhanced at lower pH) and significantly boosting antibacterial efficacy [47]. These examples underscore the potential of MOFs to overcome challenges like drug degradation, burst release, and targeted delivery in antimicrobial therapy.

#### 4.3.3 Nucleic acid therapeutics

MOFs demonstrate significant promise for nucleic acid delivery due to their tunable porosity and biocompatibility. Yang et al. developed theranostic Ce6-DNAzyme@ZIF-8@PEG nanoparticles (CDZP NPs), which not only facilitated mRNA delivery but also suppressed ROS scavenging via GPX4-DNAzyme to amplify photodynamic therapy (PDT) efficacy. This dual functionality achieved a substantial tumor suppression rate of 72.3% in breast cancer models, underscoring MOFs' capacity to synergize gene delivery with adjuvant therapies [109]. Further highlighting MOFs' versatility in precision genome editing, Pu et al. engineered ultrasound-responsive P/M@CasMTH1 nanoparticles for the cluster regularly interspaced short palindromic repeat (CRISPR)-associated protein 9 (Cas9) system. These particles generated ROS under ultrasonic stimulation, cleaved disulfide bonds to release CRISPR-Cas9 spatiotemporally, and augmented sonodynamic therapy (SDT) for synergistic tumor eradication [110]. These advances suggest that MOF-based platforms could overcome key challenges in nucleic acid delivery, including poor stability and off-target effects, while enabling real-time therapeutic monitoring.

### 4.4 Analysis of drug structure

The application of MOFs in drug structure elucidation is primarily realized through two innovative methodologies: the Crystalline Sponge Method and Coordinative Alignment (CAL). These approaches fundamentally address a major bottleneck in structural pharmacology—the traditional requirement for single-crystal growth of target molecules for X-ray analysis—by using MOFs as pre-structured host matrices that can immobilize and orderly arrange drug molecules, even those resisting crystallization.

The Crystalline Sponge Method, first reported by the Fujita group in 2013, operates by exposing MOF crystals to a solution containing the target drug molecules [111]. The guests diffuse into the regular nanochannels of the MOF and become fixed in position. Since the MOF itself forms a highly ordered crystalline framework, the entrapped molecules adopt periodic arrangements, enabling their three-dimensional structures to be resolved via conventional SXRD.

In a complementary strategy, Yaghi's team developed the CAL method using chiral MOF-520, successfully determining the crystal structures of 16 different molecules [112]. A key advantage of CAL is the potential formation of coordinative bonds between the guest molecules and the MOF scaffold, which significantly reduces molecular motion within the pores and leads to higher-resolution structural data. The power of this technique is evident in its capability to discern subtle stereochemical differences and even resolve chiral molecules.

Most recently, the integration of microcrystal electron diffraction (MicroED) with MOF-based encapsulation has further revolutionized the field by dramatically reducing sample requirements [[113], [114], [115]]. This combination now allows for accurate structural determination of pharmaceutically relevant compounds, including those available only in miniscule quantities, thereby expanding the toolbox for drug discovery and development.

### 4.5 Advanced functional MOFs

#### 4.5.1 Stimuli-responsive MOFs

**Fig. 5:**
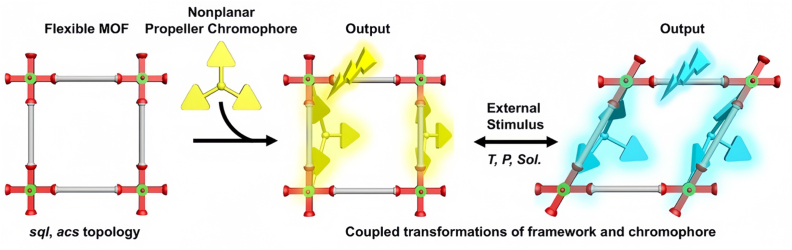
Illustration of the luminescence tuning of the organic chromophore grafted in the dynamic coordination space of a flexible MOF. The square distortion represents the space transformation induced by external stimuli (e.g., temperature, pressure, solvent), which acts as a “secondary stimulus” to trigger the conformational variations of the attached organic chromophore, leading to the change of output luminescence signal. *Adapted with permission from Z.Q. Yao et al., Angewandte Chemie International Edition, 2022, 61(17), e202202073. Copyright 2022 Wiley-VCH GmbH.*

Researchers have developed new materials that are sensitive and reversible in response to various stimuli, such as light, heat, force, solvents and chemical vapors, through ingenious molecular design. Wang et al. developed a Cd-MOF (Cd-tcbpe) based on a tetraphenylethylene derivative, which exhibits reversible fluorescence color changes from cyan to yellow-green in response to mechanical force, water, small molecule vapors (e.g., NH_3_, CH_3_COOH), and temperature. This material shows promising potential for applications in anti-counterfeiting, sensing, information storage, and white LED lighting [116]. Li et al. constructed MOFs (NWM-1-3) encapsulating amethyst derivatives by using the host-guest strategy. These MOFs exhibit photochromism under ultraviolet light irradiation and have been successfully applied to high-sensitivity amine sensing and information anti-counterfeiting [117]. Yao et al. have made significant progress in the dynamic behavior regulation of flexible MOFs. By precisely controlling the structural transformation behavior of MOFs by adjusting the stiffness of the connectors, their materials possess rare “thermoforce-induced” triple fluorescence color-changing properties, and show an excellent linear relationship between the response and the stimulus, which holds great potential in high-precision sensing and display devices (Fig. 5) [118].

#### 4.5.2 Self-propelled nanomachines

Due to their tunable channels, high specific surface area and good biocompatibility, MOFs have become an ideal platform for the construction of efficient nanomotors. Liu et al. developed ZIF series of self-enabling MOF micromotors, which can generate ion gradients through self-degradation in water to achieve self-propulsion. The metal ions released have antibacterial functions, and movement can significantly enhance the efficiency of bacterial killing and accelerate the healing of infected wounds [119]. Yu et al. constructed an enzyme-driven nanomotor (GC6@cPt ZIF) with cisplatin prodrug as the framework. It uses glucose as fuel to generate oxygen to boost and enhance the drug's ability to penetrate tumors, while consuming glutathione and enhancing the efficacy of chemotherapy and photodynamic therapy, achieving efficient combined tumor treatment [120]. Guo et al. developed buoyancy-driven nanorobots based on ZF-8. After functionalizing antibodies, they can autonomously recognize, capture and enrich specific cancer cells, providing a new tool for cell isolation [121]. Huang et al. reported a large-scale self-assembly strategy based on MOF colloids for constructing bubble-propelled micromachines (MOFtors), which can efficiently remove dyes and heavy metal ions without stirring, demonstrating its potential applications in environmental remediation [122].

These studies not only deepen the understanding of the structure-activity relationship of MOFs materials, but also greatly expand their application boundaries in fields such as biomedicine, environmental remediation, intelligent sensing and anti-counterfeiting, indicating the broad prospects of MOFs materials in solving practical complex problems.

## 5 Conclusion and perspective

### 5.1 Pharmaceutical research significance

MOFs have demonstrated significant potential in the field of pharmaceutics, offering a new paradigm for the development of precision drug delivery systems due to their highly tunable pore structures, exceptionally large surface areas, and modular synthetic nature [76,89]. The structural and functional versatility of MOFs enables the rational design of carriers that can address multiple pharmacological challenges simultaneously, from enhanced loading to stimulus-responsive release.

Through precise selection of metal nodes and organic ligands, MOFs can achieve high-capacity drug loading—exceeding 50% in some systems—as well as pH-, light-, or magnetically responsive release and targeted delivery. These capabilities significantly improve the therapeutic index of various drugs, including anticancer agents and antibiotics [70]. For instance, the ZIF-8 framework enables intelligent drug release within the acidic tumor microenvironment, concurrently delivering α-tocopheryl succinate (α-TOS) and demonstrating high efficacy in suppressing tumor growth [123]. Moreover, MOFs provide a compelling strategy for enhancing the performance of challenging natural compounds, which are often limited by poor solubility and low bioavailability. Encapsulation within water-stable MOFs can markedly improve these properties, as demonstrated with compounds such as isosteviol [49]. Additionally, the porous host structure allows for sustained drug release, mitigating burst release effects and reducing systemic side effects [70]. Beyond serving as inert carriers, certain MOFs exhibit intrinsic bioactivity attributable to their metal ions. For example, zinc or iron ions released during framework degradation can induce apoptosis in cancer cells or participate in physiological processes, adding a therapeutic dimension to the carrier itself [87,124]. In the realm of pharmaceutical analysis, MOFs are also employed as advanced sorbents for the specific adsorption and enrichment of drug molecules, thereby improving the sensitivity and accuracy of detection methods.

### 5.2 Challenges

The development of MOFs faces several critical challenges. Firstly, there is an urgent need to advance greener, more efficient, and lower-cost synthesis methods while enhancing their structural stability [46,125]. Secondly, a significant concern is the potential leakage of metal ions, which may lead to excessive local tissue concentration and induce metal toxicity. This necessitates further investigation to determine the safe threshold concentrations of MOFs in different tissues [87]. Additionally, several bottlenecks in the practical application of MOFs have been identified: (1) only about 23% of studies report PXRD verification for structural validation; (2) batch variability remains high, with PCN-222 synthesis successful in only ∼20% of laboratories (3) metal ion release (e.g., Zn^2+^ from ZIF-8); must be controlled below toxic thresholds (e.g., <30 ​μg/mL); and (4) scalable production is still limited, as current microwave synthesis typically yields <10 ​g per batch [126].

### 5.3 Current preclinical progress and barriers to clinical application

Although the clinical translation of MOF-based drug delivery systems remains at an early stage, several candidates have demonstrated considerable preclinical potential. For instance, Ni et al. developed a bilayer-structured MOF (DOX@NH_2_-MIL-88B-On-NH_2_-MIL-88B) capable of pH/glutathione dual-responsive drug release, achieving a drug loading capacity of 14.4 ​wt% and an *in vitro* inhibition rate exceeding 80% against 4T1 cancer cells [127]. Similarly, paclitaxel-loaded PCN-222 (a zirconium-based MOF) delivered via a hydrogel markedly suppressed tumor metastasis in a murine pancreatic cancer model, showing over 50% higher efficacy than the free drug while maintaining stability for 12 months [128].

However, substantial barriers impede clinical application. Critical issues such as drug loading and release kinetics remain inadequately studied, and the potential clinical toxicity of MOF nanoparticles has not been comprehensively characterized—existing toxicological data are still insufficient for definitive conclusions. A major obstacle to clinical translation is batch-to-batch reproducibility. Variations in crystal size and pore size distribution between synthetic batches directly impact drug loading and release behaviors. Fluctuations in particle size distribution alter the specific surface area, leading to inconsistent drug-loading capacity (DLC). Moreover, pore size must closely match drug molecular dimensions to achieve high loading efficiency (e.g., 5-FU loading reached 44.75 ​wt%, significantly higher than the 4.79 ​wt% for 6-mercaptopurine [129]). Even minor deviations in pore size can markedly alter drug-framework interactions, resulting in poorly controlled release kinetics and irreproducible therapeutic outcomes across batches.

In essence, batch-dependent variations in particle and pore size directly cause fluctuations in DLC and undermine the controllability of release kinetics by affecting specific surface area, pore volume, and host-guest interactions. Ongoing efforts aimed at advanced synthetic monitoring, surface engineering, defect control, and composite material design seek to mitigate these challenges and facilitate the practical application of MOFs in drug delivery [[130], [131], [132]].

### 5.4 Biological safety

The biosafety of MOFs, especially the long-term toxicity and organ-specific accumulation of their degradation products, is a key consideration for clinical transformation. The toxicity of different MOFs varies significantly, mainly due to their metal ions, organic ligands, size, and stability. A comparison of the *in vivo* toxicological profiles of the principal MOF categories is presented in Table 2.

**Table 2:** Comparison of *in vivo* toxicological data for major MOF categories.

MOF Type	Metal Ion/Linker	Key In Vivo Toxicological Findings (Model, Dose)	Organ-Specific Accumulation&Long-Term Toxicity Observations	Ref
ZIF-8	Zn^2+^/2-methylimidazole	Moderate toxicity (Zebrafish); Liver swelling, vacuolation; disrupted intestinal epithelium and hepatocyte mitochondria.	Significant liver burden; Long-term risks from Zn^2+^ and organic linker require investigation.	[133]
MIL-101(Cr)	Cr^3+^/Terephthalic acid	Low acute toxicity (Mouse, oral); No mortality or adverse effects at high doses (2000 ​mg/kg); NOAEL (No Observed Adverse Effect Level) ​≥ ​1000 ​mg/kg/day in 28-day study.	Renal clearance; No significant metal accumulation observed; favorable profile for specific applications.	[134]
Fe-MILs	Fe^3+^/Carboxylate linkers	Low acute toxicity (Rat, IV); Rapid uptake and biodegradation in liver/spleen.	Degradation products excreted; No significant chronic toxicity or accumulation reported.	[135]
Cu-MOF	Cu^2+^/Various linkers	High toxicity (Zebrafish); Severe liver damage, mitochondrial disruption.	Pronounced liver accumulation; High potential for long-term hepatotoxicity due to Cu^2+^ release.	[133]
Zr-MOF	Zr^4+^/Porphyrin (e.g., PCN-224)	Size-dependent (Mouse, IV); Ultra-small nanoparticles (∼4.5 ​nm) showed efficient renal clearance.	No long-term retention; Rapid clearance reduces chronic toxicity risk. Larger particles may accumulate in the RES.	[136]

In summary, while stable or rapidly cleared MOFs like MIL-101 (Cr) and ultrasmall Zr-MOFs present lower risks, others like ZIF-8 and particularly Cu-MOFs raise significant concerns regarding chronic liver toxicity from metal accumulation, necessitating careful design for clinical translation.

### 5.5 Future directions

Looking forward, the future development of MOFs will focus on several key directions. Novel synthetic strategies are expected to yield MOFs with enhanced performance, broader functionality, and improved safety profiles. Next-generation MOF development is exemplified by machine learning-assisted design (e.g., AI-optimized MIL-101 with 40% enhanced drug loading) and biomimetic coating technologies (e.g., mesenchymal stem cell membrane-coated Ce-UiO-66 exhibiting a 3-fold prolongation in circulation time) [126,137].

Clinical advances also provide valuable insights. For instance, RiMO-301, currently in Phase II trials as a radiosensitizer for head and neck cancer, offers potential guidance for other applications including cardiovascular therapy [126,138]. Overall, these advancements and explorations will drive the further development and application of MOFs.

## CRediT authorship contribution statement

**Zimeng Tao:** Writing – original draft, Formal analysis, Conceptualization. **Kun Hu:** Data curation, Conceptualization. **Baoxi Zhang:** Formal analysis, Data curation. **Shiying Yang:** Formal analysis, Data curation. **Dezhi Yang:** Writing – review & editing, Methodology, Funding acquisition. **Zhehui Zhao:** Methodology, Conceptualization. **Li Zhang:** Methodology. **Yang Lu:** Methodology, Funding acquisition.

## Ethics approval

Not applicable.

## Declaration of generative AI in scientific writing

During the preparation of this work, the authors used DeepSeek to improve language and readability. After using this tool, the authors reviewed and edited the content as needed and take full responsibility for the content of the publication.

## Funding information

This research was funded by the 10.13039/501100001809National Natural Science Foundation of China [Grant No. 22278443] and the CAMS Innovation Fund for Medical Sciences [Grant No. 2022-I2M-1–015].

## Data availability

Not applicable.

## Declaration of competing interests

The authors declare that they have no known competing financial interests or personal relationships that could have appeared to influence the work reported in this paper.
